# Clustering of transcriptional profiles identifies changes to insulin signaling as an early event in a mouse model of Alzheimer’s disease

**DOI:** 10.1186/1471-2164-14-831

**Published:** 2013-11-25

**Authors:** Harriet M Jackson, Ileana Soto, Leah C Graham, Gregory W Carter, Gareth R Howell

**Affiliations:** 1 The Jackson Laboratory, 600 Main Street, Bar Harbor, Maine 04609, USA; 2 School of Medicine, Tufts University, Boston, USA

## Abstract

**Background:**

Alzheimer’s disease affects more than 35 million people worldwide but there is no known cure. Age is the strongest risk factor for Alzheimer’s disease but it is not clear how age-related changes impact the disease. Here, we used a mouse model of Alzheimer’s disease to identify age-specific changes that occur prior to and at the onset of traditional Alzheimer-related phenotypes including amyloid plaque formation. To identify these early events we used transcriptional profiling of mouse brains combined with computational approaches including singular value decomposition and hierarchical clustering.

**Results:**

Our study identifies three key events in early stages of Alzheimer’s disease. First, the most important drivers of Alzheimer’s disease onset in these mice are age-specific changes. These include perturbations of the ribosome and oxidative phosphorylation pathways. Second, the earliest detectable disease-specific changes occur to genes commonly associated with the hypothalamic-adrenal-pituitary (HPA) axis. These include the down-regulation of genes relating to metabolism, depression and appetite. Finally, insulin signaling, in particular the down-regulation of the insulin receptor substrate 4 (*Irs4*) gene, may be an important event in the transition from age-related changes to Alzheimer’s disease specific-changes.

**Conclusion:**

A combination of transcriptional profiling combined with computational analyses has uncovered novel features relevant to Alzheimer’s disease in a widely used mouse model and offers avenues for further exploration into early stages of AD.

## Background

Alzheimer’s disease (AD) is an age-related neurodegenerative disease characterized by selective dysfunction and loss of neurons in specific regions of the brain including the cortex and hippocampus [1-3]. The levels of neuronal dysfunction correlate with learning and memory deficits that severely impede a patient’s ability to live independently. Amyloid precursor protein (APP) and its derivatives (forms of β-amyloid, Aβ) are thought to play a central role in AD, affecting both intra- and extra-neuronal processes [4-8]. With an aging population, AD is on the increase and could affect more than 50 million people worldwide by 2050 [9,10]. Attempts to develop new treatments for AD have proven unsuccessful [11-16] which likely highlights the lack of understanding of the disease, particularly its onset and early progression.

AD is commonly divided into two subtypes – early onset (or familial) and late onset (or sporadic) [17-21]. Early-onset AD generally presents itself before age 65 with late-onset AD developing later in life. Mutations identified in genes such as *APP* and presenilin 1 and 2 (*PSEN1* and *PSEN2*) contribute to early onset AD [22-25]. Variations in many genes including apolipoprotein E (*APOE*) have been associated with late-onset AD, but their interactions to cause AD are unclear [26-28]. Key hallmarks of both types of AD are neurofibrillary tangles and Aβ plaques [3,29,30]. However, their appearance, particularly neurofibrillary tangles, are likely to represent stages of the disease at which neuronal dysfunction has already begun, and it may be difficult to develop treatments that effectively target events from these stages onwards. Therefore it is essential to better understand the earlier stages of the disease that precede the onset of neurofibrillary tangles and plaques. These earlier stages are likely to extend over many decades in humans and targeting them provides the greatest opportunity for therapeutic intervention.

Identifying the onset and early progression of any complex age-related disease using humans or non-human primates alone is particularly challenging. Therefore, an increased understanding of the molecular and cellular processes occurring during early disease stages requires the use of animal models. The mouse has been widely used to investigate sub-phenotypes of AD such as amyloidosis [31-33], reactive astrogliosis [34,35] and neuroinflammation [36-38]. However, the mouse models have not been used to identify the molecular events that occur very early during disease, prior to or at the onset of the traditional hallmarks of AD. To identify these early molecular changes we performed clustering of transcriptional profiling data generated from mouse brains that showed a range in disease severity from no to early signs of AD. We had previously applied transcript clustering to identify early molecular stages in a mouse model of glaucoma [39-41]. In this present study, we used the *APP*^*swe*^*Psen1*^*de9*^ mouse model of AD, which is reported to develop plaques from 6 months of age [2,42-44]. Transcript clustering identifies age-specific changes as critical for the onset and early progression of AD in these mice. Moreover, we used clustering of transcriptional profiles to identify changes to insulin signaling as an important early event in AD.

## Methods

### Mouse strains and husbandry

C57BL/6J.*APP*^*swe*^*Psen1*^*de9*^ mice (JR005864, [2], herein referred to as B6.*APB*^*Tg*^) were obtained from The Jackson Laboratory and maintained in 14/10-hour light/dark cycle. All experiments were approved by the Animal Care and Use Committee at The Jackson Laboratory. To generate experimental mice, B6.*APB*^*Tg/+*^ mice were mated to C57BL/6 J (B6) mice to generate AD and wild-type (WT) cohorts. To minimize gene expression variation between mice, all mice in experimental cohorts were bred in the same mouse room, were aged together (to the extent possible) and only females were assessed. Mice from 2–12 months old were used in this study.

### Tissue harvesting, RNA isolation and sequencing

#### Tissue harvesting

A total of 22 mice were selected for transcriptional profiling: 8 at 4 months (4 WT, 4 AD), 7 at 5 months (all AD) and 7 at 6 months (3 WT, 4 AD). At the above ages, mice were sacrificed and the brain dissected free from the skull. The left hemisphere was snap frozen for preservation of RNA for gene expression studies. The right hemisphere was fixed in 4% paraformaldehyde overnight at 4°C and stored in 1× Phosphate-buffered solution (PBS) at 4°C for future use.

#### RNA isolation and library preparation

RNA was extracted from the left hemisphere of each brain using Trizol (Invitrogen, CA). mRNA was purified from total RNA using biotin-tagged poly dT oligonucleotides and streptavidin-coated magnetic beads followed by QC using an Agilent Technologies 2100 Bioanalyzer. The mRNA was then fragmented, and double-stranded cDNA generated by random priming. The ends of the fragmented DNA were converted into phosphorylated blunt ends. An ‘A’ base was added to the 3’ ends. Illumina®-specific adaptors were ligated to the DNA fragments. Using magnetic bead technology, the ligated fragments were size-selected and then a final PCR was performed to enrich the adapter-modified DNA fragments, since only the DNA fragments with adaptors at both ends will amplify.

#### High-throughput sequencing

To minimize sequencing batch effects, all 22 samples were barcoded and combined, and sequenced across six lanes on an Illumina HiSeq 2000 using standard conditions to generate 100 bp paired end sequences. A minimum of 29 million paired end reads were generated for each sample.

### RNA Sequence analysis and clustering

Analysis of sequence was performed in a private instance of the publicly available Galaxy (https://main.g2.bx.psu.edu/).

#### Quality control of sequence

Sequence quality was assessed using Fastqc QC (v0.5, Babraham). Results showed that the first 16 bases showed minor sequencing bias so these were trimmed from the sequencing reads. After trimming, the average quality score at each base position was greater than 30 (with the majority being closer to 40).

#### Alignment of Sequence

Tophat/Bowtie (v1.5.0) was used to align sequences to the mouse genome (assembly NCBI37). Flagstat (v1.0.0) was used to show that at least 74.9% of pair end reads aligned to the mouse genome.

#### Determining FPKM

For each sample, fragment length per kilobase of exon per million fragments mapped (FPKM) values were generated using Cufflinks (v1.3.0). Quartile normalization (removal of the top 25% of genes from the FPKM denominator) and bias correction (to improve accuracy of transcript abundance estimation) were used. Mouse transcripts were taken from the NCBIM37.59 gene set.

#### Determining differentially expressed genes

Differentially expressed genes were determined using Cuffdiff (v0.0.5). Again, quartile normalization and bias correction were used.

### Computational analyses

#### Singular value decomposition and hierarchical clustering

For each data subset, aligned paired-end reads were filtered to remove any gene that was not expressed in any of the samples. For the 15 samples at 4 and 6 months, filtering yielded 16888 genes. The profile of each gene was centered on the mean of WT samples at 4 months. Hierarchical clustering was performed based on Euclidean Distance between genes across all samples to identify overall similarity. Singular value decomposition (SVD) was used to identify common signals and verify consistency across replicate cohorts. The first SVD component of the 15 samples at 4 and 6 months, which separated the two age cohorts, accounted for 38% of the global variance. The second component, primarily driven by mutant samples at 4 months, represented 10% of the variance. For the subset of 8 samples taken at 4 months, the first SVD component separated mutant from WT samples and accounted for 28% of the variance.

#### Pathway analyses of differentially expressed genes

Annotations were generated and overrepresented pathways were identified using DAVID v6.7 (http://david.abcc.ncifcrf.gov/). P values reported in this study were corrected for multiple tests. Pathways were colored using Kyoto Encyclopedia of Genes and Genomes (KEGG - http://www.genome.jp/kegg/tool/map_pathway2.html).

### Immunofluorescence, immunohistochemistry and imaging

#### Immunofluorescence

Mice were transcardially perfused with 4% PFA in 1× PBS. Brains were dissected and postfixed in 4% PFA overnight, cryoprotected in 10% and 30% sucrose, and embedded in OCT. Frozen sections (25 μm) were incubated for 1–2 nights in the following primary antibodies: mouse anti-phosphorylated neurofilament (2 F11; 1:1,000; Dako), rabbit anti-IBA1 (1:500, Wako), rabbit anti-MBP (1:1000; Abcam), rabbit polyclonal GFAP (1:300, Dako); rabbit polyclonal OXT (1:200, Abcam); and goat polyclonal IRS4 (1:200; Everest Biotechnology). With the exception of anti-OXT, the rabbit polyclonal antibodies were diluted in PBTB (1× PBS with 1% TritonX-100 and 1% BSA) containing 10% of normal goat serum and the goat polyclonal antibody was diluted in PBT (1× PBS with 1% TritonX-100) containing 10% donkey serum. For anti-OXT, the BSA was not included in the dilution buffer. After incubation with the primary antibodies, the brain sections were washed with PBT and incubated in the respective secondary antibodies (goat anti-mouse Alexa Fluor 647 and goat anti-rabbit Alexa Fluor 488, 1:1000 dilution, Invitrogen) for 2 h, washed in PBT, counterstained with DAPI, and mounted with Aqua-PolyMount. For Thio-T staining, brain sections processed for GFAP immunohistochemistry were incubated 10 min in 1% Thio-T (diluted in dH_2_O), transferred to 0.5% Acetic acid for 10 min, rinsed in dH_2_O for 5 min and mounted with Aqua-PolyMount. Thio-T is a dye that binds to beta sheet-rich structures such as amyloid plaques and produces similar results to those obtained with antibodies to Aβ such as 4G10 and 6E10 (data not shown).

#### Imaging

For each antibody, at least 4 AD and 4 WT sections from multiple brain regions were assessed. Particular attention was given to the cortex, hippocampus and hypothalamus. Imaging and photography was performed on both a Zeiss Axio Imager microscope and a Leica SP5 confocal microscope. Post image processing was performed in Fiji (http://fiji.sc/Fiji).

## Results

### AD phenotypes first occur in B6.APB^Tg^ mice from 4 months of age

**Figure 1:**
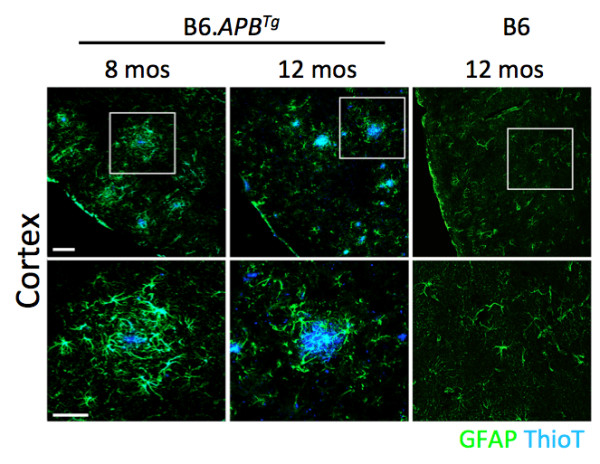
**Brain regions of female B6.*APB***^***Tg ***^**mice show amyloid plaques at 8–12 months of age.** Brains of female B6.*APB*^*Tg*^ mice were assessed for plaque deposition from 8–12 months of age. Plaques were visualized using Thioflavin T (Thio T, blue). Plaques were very obvious in the cortex, hippocampus and other brain regions in all mice assessed. Size and number of plaques increased with increasing age. In general, plaques were associated with reactive astrocytes (as determined by glial fibrillary acidic protein (GFAP) staining, green). DAPI was used to identify nuclei. Scale bars; Upper panels = 100 μm, lower panels = 50 μm.

**Figure 2:**
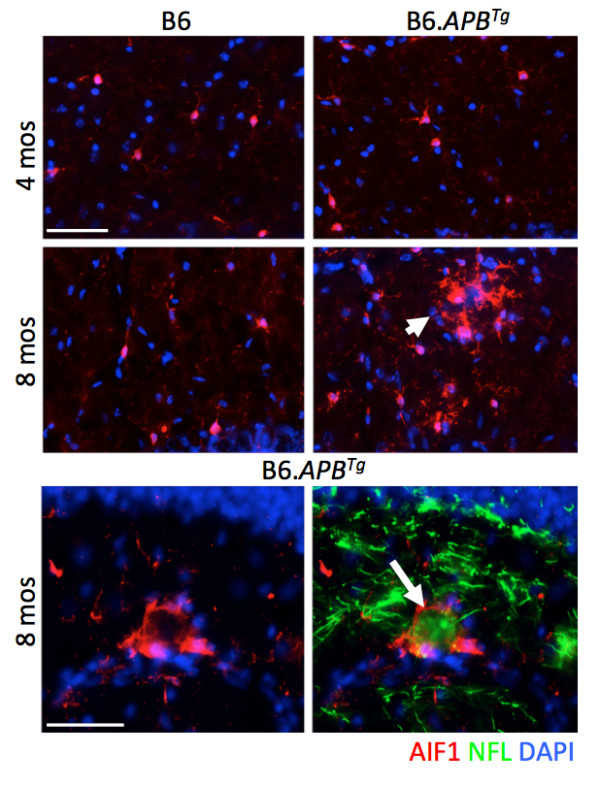
**Activation of microglia (microgliosis) coincides with focal axonal swellings in B6.*APB***^***Tg ***^**mice.** Little to no microgliosis (as judged by an increase in AIF1, red) was observed in B6.*APB*^*Tg*^ mice at 4 months but significant levels of microgliosis were observed in multiple brain regions at 8 months of age (arrowhead). Images taken from a region of the hippocampus as an example. AIF1 (allograft inducible factor, formerly IBA1) is a marker of microglia. Occasionally, this coincided with localized regions of axonal swellings (arrow, visualized using neurofilament, NFL, green). DAPI was used to identify nuclei. Scale bars = 50 μm.

**Figure 3:**
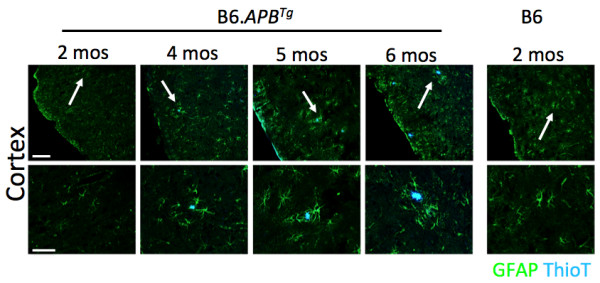
**AD phenotypes first appear in female B6.*APB***^***Tg ***^**mice between 4–6 months of age.** To determine the ideal ages at which to perform gene expression profiling we assessed plaque formation in brains from female B6.*APB*^*Tg*^ mice between 2–6 months of age. Plaques were visualized using Thioflavin T (Thio T, blue). The earliest detectable plaques occurred in the cortex of 4 months old mice. No plaques were observed in 2 months old mice. As expected, plaques were more apparent in brains from 6 months old mice. Arrows in the upper panels indicate the region shown in the lower panels. Scale bars; upper panels = 100 μm, lower panels = 50 μm.

The main aim of this study was to use transcript profiling to identify molecular changes that impact the onset and very early stages of AD. Therefore, to determine the ideal ages to profile, we assessed B6.*APB*^*Tg*^ female mice between 2 and 12 months of age for AD phenotypes including plaque formation, reactive astrogliosis and activation of microglia (Figures 1, 2 and 3).

Previous reports using the B6.*APB*^*Tg*^ strain show that females develop plaques and other AD-like phenotypes from around 6–8 months of age [2,42-44]. Given the potential for environmental differences to influence onset and progression of AD in different colonies of mice, we first assessed AD phenotypes in our colony from 8–12 months of age. As expected, mice of all ages within this span showed plaques in multiple brain regions and there was an observable increase in plaque numbers and size with increasing age (Figure 1). Activation of microglia both surrounding plaques and associated with axonal swellings was also observed at these time points (Figure 2). As previously reported, we saw no significant loss of neurons in any brain regions including the cortex and hippocampus at these ages (data not shown). These results indicate that onset and early stages of AD are occurring in B6.*APB*^*Tg*^ female mice younger than 8 months old.

Therefore, we assessed AD phenotypes in mice 2–6 months old. At 2 months of age, B6.*APB*^*Tg*^ female mice were indistinguishable from WT controls. However, by 4 months of age, very small plaques were observed in discrete regions of the brain, particularly in the cortex (Figure 3). Plaques were readily observed in 6 months mice in the cortex and hippocampus. Together these results indicated that profiling B6.*APB*^*Tg*^ female mice at 4–6 months of age would identify very early molecular changes that may be key drivers and/or important biomarkers of early stages of AD.

### Clustering of transcriptional profiles identifies age is a major driver of disease onset

**Figure 4:**
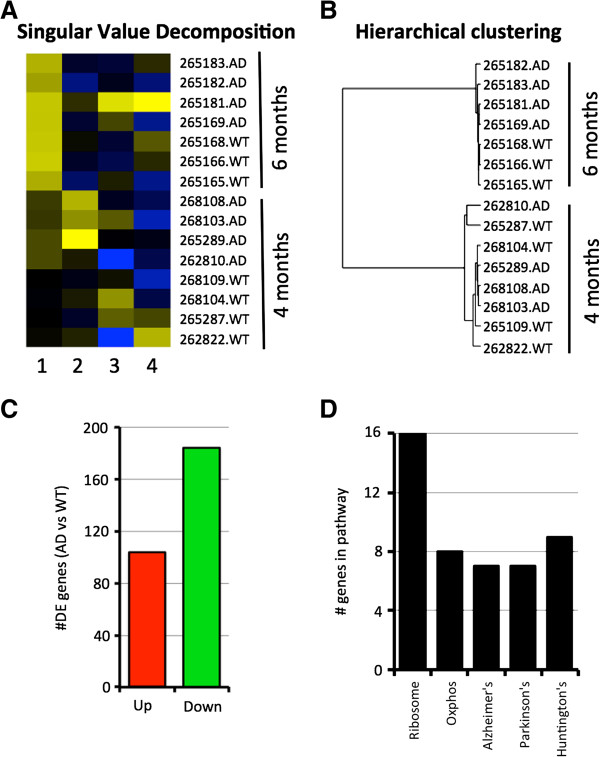
**Transcript clustering implies that age-related changes are of primary importance in the development of AD phenotypes. (A)** Singular value decomposition of 4 and 6 months samples shows that age (column 1) is the greatest driver of variation between samples. Column 2 shows that genotype is the second greatest driver of variation. **(B)** This is confirmed using hierarchical clustering. **(C)** Comparing the 4 months samples with the 6 months samples using Cuffdiff identified 288 genes that were differentially expressed (184 upregulated, 104 down regulated, see Additional file 1: Table S1). **(D)** Pathway analysis using DAVID showed that members of the ribosome and oxidative phosphorylation pathways (from KEGG) are significantly overrepresented (P < 0.05) in the DE gene list.

To identify early molecular changes in AD, we used RNA-seq to generate transcriptional profiles from 22 female mice (15 AD and 7 WT) from 4, 5, and 6 months of age (see Methods). For each sample, reads were aligned using Tophat [45] and normalized FPKM values were generated for every expressed gene using Cufflinks [46]. To determine the processes driving variation between all 4 and 6 months samples, we used singular value decomposition (SVD). Genes that were not expressed in any samples were removed from the analyses. Strikingly, the largest component of variation was age as the greatest separation was observed between the 4 and 6 months samples (irrespective of genotype). Genotype (i.e. carrying the AD transgenes) was the second major component (Figure 4A). Hierarchical clustering of the samples showed a similar outcome (Figure 4B). Therefore, key age-specific changes are likely to occurring at these ages to drive the onset and early progression of AD phenotypes.

To identify these age-specific changes responsible for the variation between the 4 and 6 months samples, we compared transcript profiles from all 4 months samples (4 AD and 4 WT, 8 in total) to profiles from all 6 months samples (4 AD and 3 WT, 7 in total) using Cuffdiff (see Methods). A total of 288 genes were differentially expressed (DE) between the ‘all 4 months’ and ‘all 6 months’ groups (Figure 4C and Additional file 1). Similar results were obtained when comparing either the 4 months AD samples to the 6 months AD samples, or the 4 months WT to the 6 months WT samples (data not shown) indicating that these truly are age-specific changes. Pathway analysis revealed that the ribosome and oxidative phosphorylation pathways were over-represented in the 288 DE genes (Figure 4D). These pathways have previously been linked to aging including down-regulation of protein synthesis/mRNA processing and mitochondrial dysfunction [47-54]. Our data imply that perturbations in these pathways are necessary for the onset or early progression of AD in B6.*APB*^*Tg*^ mice. Understanding these age-specific changes is likely important for all forms of Alzheimer’s disease, not just the familial form modeled in B6.*APB*^*Tg*^ mice.

### Disruption to genes normally associated with the hypothalamic-pituitary-adrenal (HPA) axis is an early event in B6.APB^Tg^ mice

**Figure 5:**
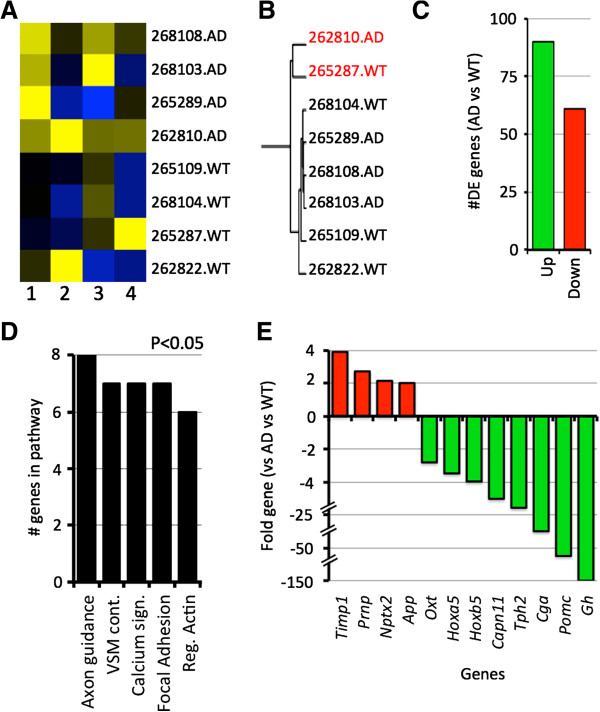
**Transcript clustering identifies that genotype is the major driver of variation in the 4 months samples. (A)** Singular value decomposition stratifies the 4 months samples into two groups based on genotype. **(B)**. In addition, hierarchical clustering, suggests that two samples (shown in red) are outliers compared to the other samples, and so are not included in comparisons to identify differentially expressed (DE) genes. **(C)** DE genes were identified by comparing the three 4 months B6.*APB*^*Tg*^ (AD) samples with the three B6 control samples (WT) using Cuffdiff. A total of 151 DE genes were identified (90 upregulated, 61 downregulated, see Additional file 2: Table S2). **(D)** Pathway analysis identified axon guidance, vascular smooth muscle contraction (VSM cont.) and calcium signaling (Calc sign.) as over-represented pathways (P < 0.05). **(E)** Analysis of the DE genes with the greatest fold changes identified genes commonly associated with the hypothalamus as down regulated including oxytocin (*Oxt*), glycoprotein hormones, alpha polypeptide (*Cga*), pro-opiomelanocortin-alpha (*Pomc*) and growth hormone (*Gh*).

After age, SVD identifies AD genotype as the second greatest component of variation in the 8 samples from the 4 months group (Figure 5A). This is the age at which AD phenotypes were first observed (Figure 3). However, hierarchical clustering identifies that 2 samples (1 AD and 1 WT) are outliers and likely to decrease the sensitivity to detect true AD-relevant changes (Figure 5B). Therefore, to identify DE genes important for AD onset we compared the transcript profiles of the 6 most closely related 4 months samples (3 AD samples vs 3 WT samples) using Cuffdiff. A total of 151 genes were DE between the 4 months AD and WT groups (Figure 5C and Additional file 2).

**Figure 6:**
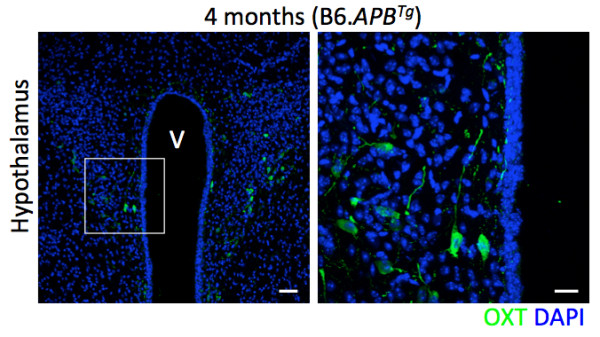
**Oxytocin (OXT - green) is localized to neurons in the hypothalamus, particularly surrounding the third ventricle (V).** OXT was not observed in all other brain regions assessed (data not shown). Nuclei are visualized with DAPI (blue). Scale bars: left panel = 50 μm, right panel = 20 μm.

Six of the most downregulated genes comparing the 4 months AD and WT groups are hormones or hormone-related. These six genes include growth hormone (*Gh*, -137.6×), pro-opiomelanocortin (*Pomc*, -5.5×) and oxytocin (*Oxt*, -2.8×). Interestingly, these six genes are commonly associated with the HPA region of the brain and transcript analysis (available through the Allen Brain Atlas) shows expression for *Oxt* and *Pomc* only in the hypothalamus. Our data shows similar findings; with the OXT protein almost exclusively localized to the hypothalamic regions surrounding the third ventricle (Figure 6). These HPA-associated genes are involved in a variety of different behaviors including depression, appetite and metabolism. These behaviors have been previously associated with human Alzheimer’s disease [55-57]. Our analyses indicate that alterations in these behaviors may occur early in AD and studying these genes may be important for developing new therapies or as diagnostic criteria for AD.

### Clustering of 5 months samples identifies perturbation in the insulin signaling pathway as an important event in AD

**Figure 7:**
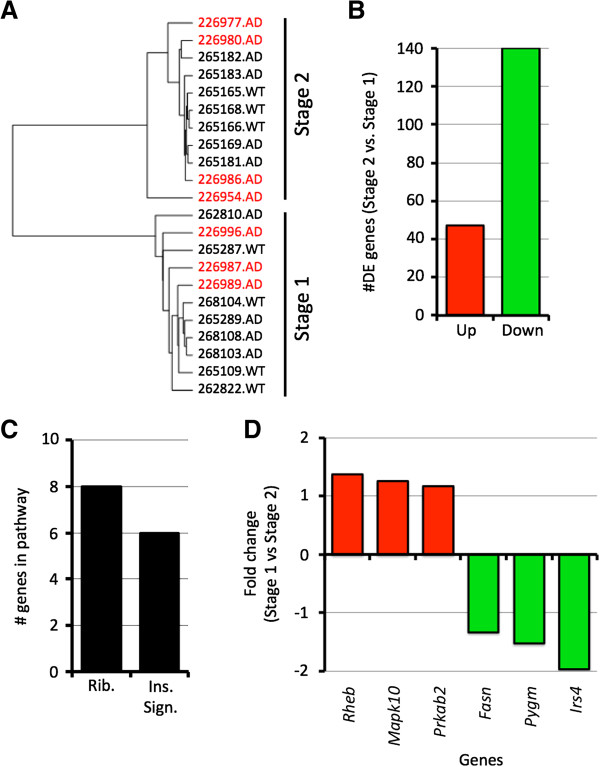
**Transcript clustering of 5 months samples identified insulin signaling as a critical pathway activated in early stages of AD. (A)** Hierarchical clustering clustered three of the 5 months AD samples with the 4 months samples (now termed Stage 1), and the remaining four with the 6 months samples (now termed Stage 2). The 5 months AD samples are shown in red. **(B)** The 5 months samples in Stage 1 were compared with the 5 months samples in Stage 2 using Cuffdiff. A total of 187 genes were differentially expressed (47 upregulated, 187 downregulated, see Additional file 3). **(C)** Pathway analysis showed that both the ribosome (Rib.) and insulin signaling pathway (Ins. Sign.) were overrepresented in the DE genes (P < 0.05). **(D)** Comparing Stage 1 with Stage 2, six genes in the insulin signaling pathway were differentially expressed (3 upregulated, 3 downregulated).

Our combined approach of histology, transcriptional profiling and computational analyses demonstrates that key events, including age-specific changes, occur between 4 and 6 months of age in B6.*APB*^*Tg*^ mice. Therefore, we hypothesized that early progression of AD is likely to be occurring at 5 months as well as at 4 months of age in these mice. To test this, we analyzed the transcriptional profiles from the 7 AD transgene carrying B6.*APB*^*Tg*^ female mice at 5 months of age. We first determined the similarity of these transcriptional profiles to the 4 and 6 months AD and WT samples using hierarchical clustering. Rather than clustering into a separate ‘age-specific’ group, the 5 months samples clustered with the 4 and 6 months samples: three of the seven 5 months AD samples clustered with the 4 months samples and four of the 5 months samples clustered with the 6 months samples (Figure 7A). This shows that indeed crucial changes are occurring independent of absolute age in AD mice at 5 months of age. These changes may be ‘age-specific’ due to the 5 months old mice aging at different rates (a widely accepted phenomenon), or ‘disease-specific’ changes independent of age, or both.

To help answer these questions and to determine the gene expression differences driving the variation between the two groups of 5 months samples (termed stage 1 and stage 2) we used Cuffdiff. A total of 187 genes were differentially expressed comparing the early to late group (Figure 7B and Additional file 3). Pathway analysis of the DE genes identified the ribosome pathway and the insulin signaling pathway as overrepresented in stage 2 compared to stage 1 (Figure 7C). The ribosome pathway was also overrepresented comparing the 4 to the 6 months data suggesting that differences in physiological aging are driving some of the variation between the chronologically-matched 5 months old samples. Insulin signaling is a novel finding that was only significant in clustering of the 5 months samples. Therefore, it is not clear whether the variation between the 5 months old samples is due in part to the mice physiologically aging at different rates, or disease-specific changes.

**Figure 8:**
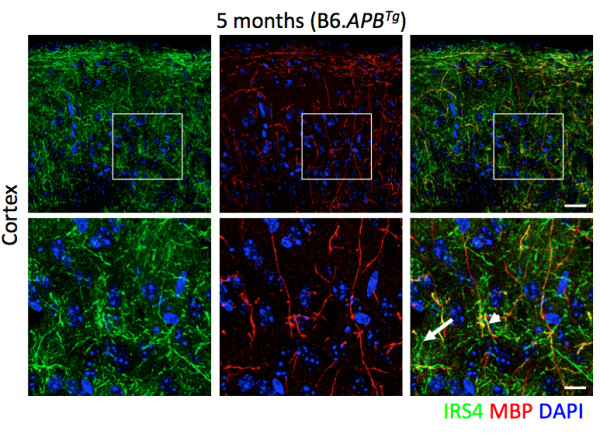
**Insulin receptor substrate 4 (IRS4) protein is localized to axons in the cortex.** Immnofluorescence was used to determine the location of IRS4 protein. The IRS4 protein (green) is readily detectable in multiple brain regions including the cortex. The majority of the IRS4 protein co-localizes with myelinated axons (as indicated by the significant overlap between IRS4 with myelin basic protein (MBP, red). Colocalization is indicated in yellow (e.g. arrowhead). However, IRS4 is also present in non-myelinated axons (longer arrow, lower right panel). Lower panels are a higher magnification of the boxed regions indicated in the upper panels. Scale bars: upper panels = 25 μm, lower panels = 10 μm.

At least six genes in the insulin signaling pathway are differentially expressed in stage 2 compared to stage 1 (Figure 7D). These include three upregulated genes (*Prkab2* 1.2×, *Rheb*, 1.3× and *Mapk10*, 1.3×) and three downregulated genes (*Fasn -*1.32×, *Pygm -*1.5× and *Irs4 -2.0*). Insulin receptor substrate 4 (*Irs4*) is the most downregulated gene in the insulin signaling pathway and is of particular interest as *Irs* genes have been shown to impact phosphorylation of tau, a crucial step in the generation of neurofibrillary tangles [58,59]. Previous studies have localized *Irs4* to specific sets of neurons in the hypothalamus [60,61] and Allen Brain Atlas for mouse shows expression of *Irs4* mainly in the hypothalamus and at lower levels in pallidum. Interestingly, the human Allen Brain reports expression of *IRS4* in both the hypothalamus and cortex. Our analysis of *Irs4* expression agrees with these findings with strongest expression in the hypothalamus and possible low-level expression in the cortex (data not shown). To confirm this we localized the IRS4 protein using an anti-IRS4 antibody (see Methods). IRS4 is indeed present in axons of neurons in both the hypothalamus and the cortex (Figure 8). Given our previous findings of early changes to genes associated with the hypothalamus, it is possible that the downregulation of *Irs4* (and other insulin signaling genes) is occurring in the HPA region, but it is also possible changes are occurring in the cortex directly and these may be key to the onset/progression of Alzheimer’s disease in these mice.

## Discussion and conclusions

Here we report the use of transcript clustering to identify molecular mechanisms contributing to early stages of AD in mice. This is the first time this approach has been used to assess early events in AD. We show that plaques first appear in the cortex of female B6.*APB*^*Tg*^ mice around 4 months, and more significantly at 6 months of age. Transcript clustering identified age as the greatest driver of variation in the 4 and 6 months samples, with genotype being the second major driver. Pathway analysis of the genes differentially expressed between the 4 and 6 months samples suggests at least two biological processes are driving these changes namely mRNA processing/protein translation and oxidative phosphorylation. Both of these processes have previously been implicated in age-related changes [47-54]. Although it is unexpected that these aging-linked changes occur so early, it may suggest that subtle aging changes occur in mice as early as 4–6 months. At this age, mice are considered to be equivalent in age to early adulthood in humans (e.g. between 20–40 years of age) an age when mild cognitive impairment (MCI) can occur in some individuals [62].

The earliest AD-relevant changes detected in 4 months AD mice compared to WT mice were hormone-related and may indicate changes to the HPA axis. Our study is the first to propose that these may be among the earliest changes in AD and that they may be important indicators of susceptibility to and the onset of AD. Genes expressed by cells in the HPA axis control behaviors such as mood, sleep, and eating and these behaviors are commonly disrupted in AD patients. For instance, systemic metabolic changes as well as mood changes have been observed in very early phases of AD and suggest early dysfunction in the hypothalamus [55-57]. Another change seen in AD patients is a behavioral one, affecting mood and depression [63-66]. Oxytocin, often prescribed as a medication to treat depression, is almost exclusively expressed in the hypothalamus [67] and our data shows a downregulation early in B6.*APB*^*Tg*^ mice.

It is not clear why the hypothalamus may be more susceptible than other brain regions to early dysfunction in AD but it may be due to the absence of a traditional blood brain barrier in this region. Instead, vessels surrounding the hypothalamus, particularly the median basal region of the hypothalamus, are fenestrated to allow the easy passage of hormones into the circulation [68-70]. This structure is constitutively leaky and may be particularly susceptible to increases in AD-relevant phenotypes such as amyloid accumulation. Furthermore, vascular dysfunction (such as cerebral hemorrhages or micro-bleeds) may precede the molecular changes identified in our study. Age-related vascular dysfunction has been reported but more detailed studies relating to the role of age-related vasculature dysfunction in AD are required.

Transcript clustering of the 5 months data identified changes to the insulin signaling pathway including *Irs4* as an early event in AD. Insulin signaling is strongly associated with aging and age-related diseases including obesity and diabetes, conditions that have recently been identified as possible risk factors for AD [71-75]. *Irs4* is expressed strongly in the hypothalamus but at lower levels in other brain regions such as the cortex. Therefore, downregulation of *Irs4* likely impacts both the hypothalamus and the cortex. IRS4 is a good candidate for further studies as it is known to function in a number of processes relevant to AD including interacting with endosomes [76] to control Aβ levels in neurons [8,77,78], or through interactions with *Irs2*[79] in the phosphorylation of *Tau* during the formation of neurofibrillary tangles [58,59]. Although further work is required to elucidate the precise role of IRS4 in AD, our data suggests that understanding the role of IRS4 in AD may lead to potential new therapies for AD.

Our study, using a genetic model relevant to early onset AD, shows that clustering of transcriptional profiling data is a powerful method to identify important early molecular changes at the onset of traditional hallmarks of AD. However, it is widely accepted that these models do not recapitulate all aspects of AD including hyperphosphorylation of Tau leading to neurofibrillary tangles and neuronal cell loss. This is intriguing given that mutations that disrupt *APP* processing do cause neurofibrillary tangles and associated neuronal changes in human AD. Given this, our work highlights the need for further investigation of B6.*APB*^*Tg*^ mice at the onset of plaque formation to better understand the role of insulin signaling and other early molecular events that follow APP misprocessing. Furthermore, our study focuses on a mouse model relevant to early onset AD, which accounts for <5% of all cases human of AD. It is not clear whether mechanisms that lead to plaque formation and neurofibrillary tangles in early onset AD are similar or distinct to those that lead to AD in late-onset AD. Nevertheless, understanding and targeting these early events is likely to provide the greatest potential for developing new treatments for Alzheimer’s disease.

### Availability of supporting data

Raw data is being made available through Geo datasets.

## Competing interests

The authors declare that they have no competing interests.

## Authors’ contributions

GRH designed and implemented the study. HMJ, IS and LCG performed the histological analyses. HMG and GRH performed the RNA-seq data analyses. GWC performed the computational analyses including singular value decomposition and hierarchical clustering. GRH wrote the manuscript and it was critically read and approved by all authors.

## Supplementary Material

Additional file 1: Table S1 Differentially expressed genes comparing 4 months with 6 months samples. Click here for file

Additional file 2: Table S2 Differentially expressed genes comparing 4 months WT with 4 months AD samples. Click here for file

Additional file 3: Table S3 Differentially expressed genes comparing Stage 1 with Stage 2 samples. Click here for file
